# Isoform-specific modulation of the chemical sensitivity of conserved TRPA1 channel in the major honeybee ectoparasitic mite, *Tropilaelaps mercedesae*

**DOI:** 10.1098/rsob.160042

**Published:** 2016-06-15

**Authors:** Xiaofeng Dong, Makiko Kashio, Guangda Peng, Xinyue Wang, Makoto Tominaga, Tatsuhiko Kadowaki

**Affiliations:** 1 Department of Biological Sciences, Xi'an Jiaotong-Liverpool University, 111 Ren'ai Road, Suzhou Dushu Lake Higher Education Town, Jiangsu Province 215123, People's Republic of China; 2 Division of Cell Signaling, Okazaki Institute for Integrative Bioscience, National Institutes of Natural Sciences, Okazaki 444-8787, Japan; 3 Department of Physiological Sciences, SOKENDAI (The Graduate University for Advanced Studies), Okazaki 444-8585, Japan

**Keywords:** TRPA1 channel, honeybee ectoparasitic mite, plant oil, honeybee decline

## Abstract

We identified and characterized the TRPA1 channel of *Tropilaelaps mercedesae* (TmTRPA1), one of two major species of honeybee ectoparasitic mite. Three TmTRPA1 isoforms with unique N-terminal sequences were activated by heat, and the isoform highly expressed in the mite's front legs, TmTRPA1b, was also activated by 27 plant-derived compounds including electrophiles. This suggests that the heat- and electrophile-dependent gating mechanisms as nocisensitive TRPA1 channel are well conserved between arthropod species. Intriguingly, one TmTRPA1 isoform, TmTRPA1a, was activated by only six compounds compared with two other isoforms, demonstrating that the N-terminal sequences are critical determinants for the chemical sensitivity. This is the first example of isoform-specific modulation of chemical sensitivity of TRPA1 channel in one species. α-terpineol showed repellent activity towards *T. mercedesae* in a laboratory assay and repressed *T. mercedesae* entry for reproduction into the brood cells with fifth instar larvae in hives. Thus, α-terpineol could be used as the potential compound to control two major honeybee ectoparasitic mites, *T. mercedesae* and *Varroa destructor*, in the apiculture industry.

## 1. Introduction

The transient receptor potential (TRP) channel family shares six common transmembrane segments that form sensor and pore domains, and confer cation permeability. However, TRP channels are unique among various ion channels by having diverse cation selectivities and activation mechanisms [1]. TRP channels play major roles for various sensory perceptions such as vision, thermosensation, olfaction, hearing, taste sensation and mechanosensation by functioning as primary signal integrators that allow animals to perceive the external stimuli [2]. TRP channels also enable individual cells to detect changes in temperature, osmolarity and fluid flow in their local environment [1,3].

The metazoan TRP family is classified into eight subfamilies—TRPA, TRPC, TRPM, TRPML, TRPN, TRPP, TRPV and TRPVL—based on the phylogenetic tree constructed by their amino acid sequences of transmembrane segments [4]. Among them, TRPA1 specifically contains 15–16 ankyrin repeats (ARs) in the N-terminus. ARs are 33 residue motifs consisting of pairs of antiparallel α-helices connected by β-hairpin motifs. ARs appear to be necessary for the sensitivity to various stimuli [5,6]. Molecular structure of TRPA1 is similar to that of TRPV1 by forming a homotetramer to contain the pore with two gates [6–8]. The physiological functions of TRPA1 have been characterized using model organisms (mouse, zebra fish, fruit fly and nematode). TRPA1 is activated by nociceptive thermal (either heat or cold) and chemical stimuli, demonstrating that it plays major roles in nociception and inflammatory pain [5]. In addition, the roles in temperature entrainment of the circadian clock, promoting longevity at cold temperatures and induction of embryonic diapause in progeny have been recently reported using *Drosophila melanogaster*, *Caenorhabtitis elegans* and *Bombyx mori*, respectively [9–11]. However, TRP channels have been characterized in a limited number of species, and we have recently identified and characterized TRPA1 channel of *Varroa destructor* (VdTRPA1), the major ectoparasitic mite of honeybee as the first parasite TRP channel [12].

The losses of managed honeybee (*Apis mellifera*) colonies have considerably increased in Europe and North America during recent years, and various pathogens and parasites associated with honeybees are considered the major causes [13]. Among the parasites, the ectoparasitic mite *V. destructor* has affected honeybees most severely*. Varroa* mites are the primary pests of honeybees in all beekeeping continents, except Australia, causing direct impacts on honeybee health (large-scale death of larvae and pupae by feeding on hemolymph) as well as indirect effects by vectoring viruses and other honeybee disease agents [13,14]. Furthermore, in many Asian countries, *A. mellifera* colonies are also infested with another ectoparasitic mite, *Tropilaelaps mercedesae*, and both mites are usually present in the single colony [15,16]. Although there are some differences in their habitats, such as shorter life cycle and phoretic phase for *Tropilaelaps* mite, they share many characteristics as new emerging parasites of *A. mellifera*. Their reproductive strategies are quite similar [16,17] and both mites were reported to vector DWV [18,19]. Thus, the negative impact of *Tropilaelaps* mite infestation on an *A. mellifera* colony is almost equal to that of the *Varroa* mite. Although the current distribution of *Tropilaelaps* mites is still limited to Asia [16], they could be spread and established in Europe and North America via global trade. The best method to control *Tropilaelaps* mites is to remove all brood for several days from the hive [16]. However, this is not practical for large-scale commercial beekeepers that manage thousands of honeybee colonies. Thus, controlling *Tropilaelaps* mites becomes as difficult as managing *Varroa* mites. Sustainable control strategies for *Tropilaelaps* mites based on the use of natural compounds would be highly desirable for beekeeping and related industries. In this study, we identified and characterized *T. mercedesae* TRPA1 (TmTRPA1) and compared it with VdTRPA1. We discuss the evolution and physiological functions of TmTRPA1, the isoform-specific modulation of chemical sensitivity and the potential use of TmTRPA1-activating compounds to control honeybee ectoparasitic mites in the apiculture industry.

## 2. Results

### 2.1. Identification of three *TmTRPA1* mRNA isoforms

**Figure 1.**
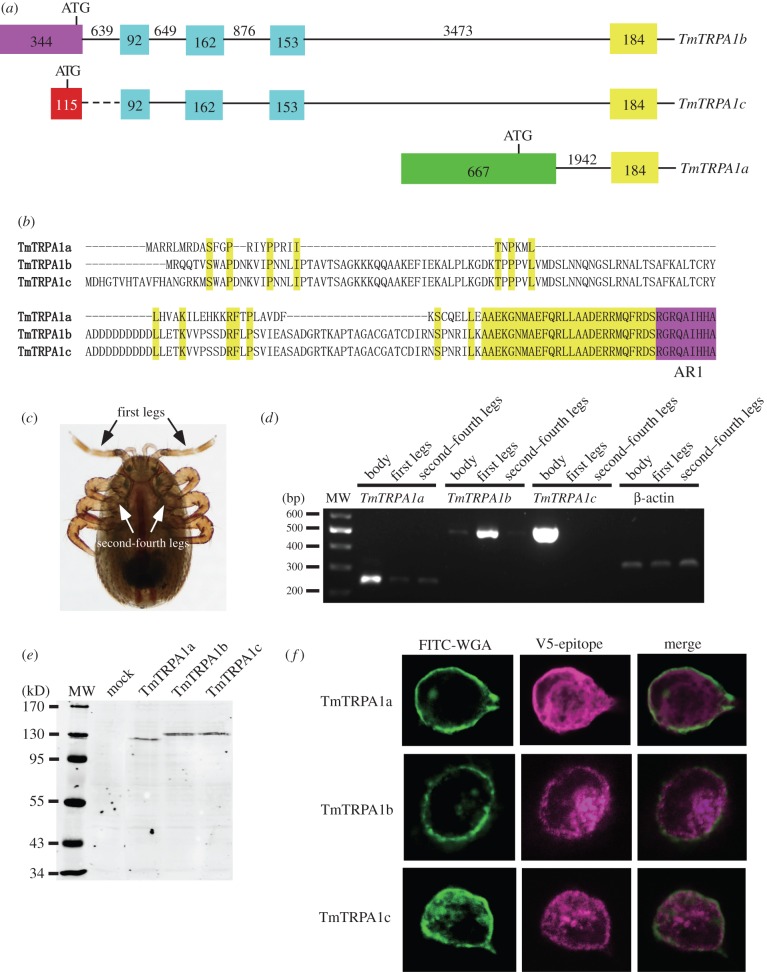
Three TmTRPA1 isoforms and their expression in HEK293 cells. (*a*) Exon–intron structures of three *TmTRPA1* mRNA isoforms, *TmTRPA1a*, *TmTRPA1b* and *TmTRPA1c*, predicted from the assembled genomic sequence of *Tropilaelaps mercedesae*. Three isoforms share the same 184 bp exon (yellow rectangle) and the downstream exons (not shown) but contain the different upstream exons. *TmTRPA1b* and *TmTRPA1c* mRNAs share the same 92, 162 and 153 bp exons (blue rectangles). Purple, red and green rectangles represent the exons unique to each isoform. The position of translational initiation codon (ATG) is also indicated for each isoform. The numbers indicate the sizes of exons and introns (lines), to and they are not to scale. The most upstream 115 bp exon (red rectangle) of *TmTRPA1c* is missing in the assembled genomic sequence and thus the size of the following intron sequence is not known (dashed line). (*b*) Alignment of the N-terminal amino acid sequences of three TmTRPA1 isoforms. The shared amino acids are highlighted with yellow and a part of the amino acid sequence of the first ankyrin repeat (AR1) is indicated with purple. (*c*) Ventral view of *T. mercedesae*. Black and white arrows indicate the first legs and the second to fourth legs, respectively. (*d*) Detection of *TmTRPA1a, TmTRPA1b, TmTRPA1c* and β-actin mRNA in the whole body without mouthparts and legs (body), the first legs and second to fourth legs by RT-PCR. The position of 200–600 bp DNA molecular weight marker (MW) is shown at the left. (*e*) Proteins expressed in HEK293 cells transfected with empty vector (mock), TmTRPA1a-, TmTRPA1b- and TmTRPA1c-expressing constructs were analysed by Western blot. The size (kD) of protein molecular weight marker (MW) is at the left. (*f*) Localizations of plasma membrane-bound FITC-WGA and either TmTRPA1a, TmTRPA1b or TmTRPA1c tagged with V5-epitope in the transfected HEK293 cells by immunofluorescence. The merged images are also shown.

We amplified a partial *TmTRPA1* cDNA at 5′ end by nested RT-PCR with degenerate primers designed based on VdTRPA1 and TRPA1 sequences of two mite/tick species, *Metaseiulus occidentalis* (XM_003748080.1) and *Ixodes scapularis* (XM_002405489.1). We obtained the RT-PCR product with an expected size of approximately 500 bp and the sequence had high similarity to TRPA1 sequences of other species. We then designed the primer sets for 5′ and 3′ RACE using this cDNA fragment, and 1.3 kb 5′ RACE and 2.3 kb 3′ RACE products were obtained and sequenced. Although only a single sequence represented the 3′ RACE product, we identified three different sequences in the 5′ RACE product, demonstrating that *TmTRPA1* mRNA has at least three isoforms. In fact, three full-length *TmTRPA1* cDNAs (*TmTRPA1a*, *TmTRPA1b* and *TmTRPA1c*) which differ at 5′ ends were finally isolated (figure 1*a*). According to the assembled genomic sequence of *T. mercedesae*, we found that these three mRNAs are encoded by 23 (*TmTRPA1a*) and 26 (*TmTRPA1b* and *TmTRPA1c*) exons, and 22 exons are shared among them. They are likely to be generated by transcription from the different initiation sites (figure 1*a*). Because the translational start codon is located in the unique 5′ end exon of each isoform, three TmTRPA1 variants contain the different N-terminal sequences. TmTRPA1b and TmTRPA1c share the most N-terminal sequence and TmTRPA1a has the shortest N-terminal sequence (figure 1*b*). Nevertheless, all isoforms contain 15 ARs and six transmembrane segments (S1–S6) forming the ion-transport domain (figure 1*b*).

### 2.2. Expression profiles of three *TmTRPA1* mRNA isoforms in *Tropilaelaps mercedesae*

We examined the expression of *TmTRPA1a*, *TmTRPA1b* and *TmTRPA1c* mRNAs in the first legs, second to fourth legs and the whole body (excluding the mouthparts and legs) of *T. mercedesae* using RT-PCR. The first legs are much longer and thinner than the other legs (figure 1*c*) and not used for mite movement, and are instead always raised in the air. This demonstrates that the first legs correspond to the insect antennae, as suggested for many mite species [20]. *TmTRPA1a* and *TmTRPA1b* mRNAs were present in all of the above body parts; however, higher levels of *TmTRPA1a* and *TmTRPA1b* mRNAs were detected in the whole body and the first legs, respectively (figure 1*d*). *TmTRPA1c* mRNA was only detected in the whole body but not in any legs by our assay (figure 1*d*). These results were reproducible with another RNA preparation. Because of the preferential expression of TmTRPA1b in the first legs of *T. mercedesae*, this isoform is likely to have major roles for sensory perception by the sensory pit organ in the first legs of mites.

### 2.3. TmTRPA1 protein expression in HEK293 cells

Prior to conducting calcium imaging of HEK293 cells expressing one of three TmTRPA1 isoforms, we characterized the protein expression and cellular localization of V5-epitope-tagged TmTRPA1 isoforms in HEK293 cells. The proteins with expected molecular weights (134, 143 and 144 kD for TmTRPA1a, TmTRPA1b and TmTRPA1c, respectively) were specifically detected using Western blot (figure 1*e*). We then tested the cellular localizations of TmTRPA1a, TmTRPA1b and TmTRPA1c by staining the cells expressing the channels with FITC-WGA (wheat germ agglutinin) and anti-V5-epitope antibody. As shown in figure 1*f*, the fractions of three isoform proteins co-localized with FITC-WGA, indicating that some of these proteins are present at the plasma membrane. TmTRPA1b isoform is highly expressed in the first legs of *Tropilaelaps* mite and localized at the plasma membrane of the transfected HEK293 cells, and it is likely to play major roles in the sensory perception. We thus focused on characterizing TmTRPA1b in this study.

### 2.4. Heat activation of TmTRPA1

**Figure 2.**
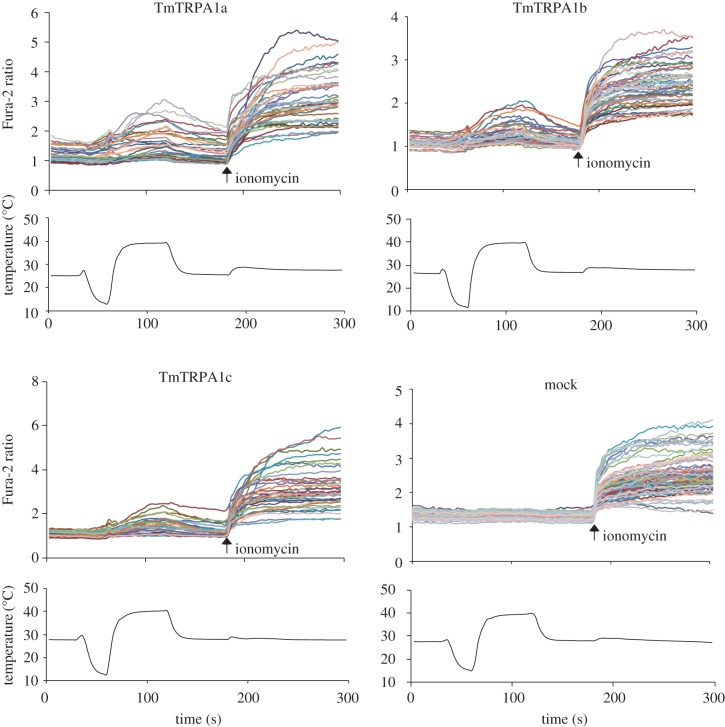
Heat activation of three TmTRPA1 isoforms. The upper traces indicate the changes of Fura-2 ratio (intracellular calcium level) in TmTRPA1a-, TmTRPA1b-, TmTRPA1c- or mock-transfected cells on temperature fluctuation in the presence of extracellular calcium. Each line represents the Fura-2 ratio in the individual cell measured by calcium imaging. The arrows show the time points when ionomycin was added. The lower traces show the changes of bath temperature by time.

We used a calcium-imaging technique with Fura-2 to measure the relative changes of intracellular calcium levels of HEK293 cells expressing TmTRPA1 by temperature fluctuations. Activation of the channel is expected to increase the intracellular calcium levels by influx of extracellular calcium. As shown in figure 2, increased temperature elevated the relative intracellular calcium levels of cells expressing TmTRPA1a, TmTRPA1b or TmTRPA1c, but not mock-transfected cells. Low temperature did not increase the relative intracellular calcium levels of cells expressing TmTRPA1 (figure 2). These results may suggest that all of TmTRPA1 isoforms are heat-activated.

### 2.5. Differential activation of TmTRPA1 isoforms by chemical compounds

**Figure 3.**
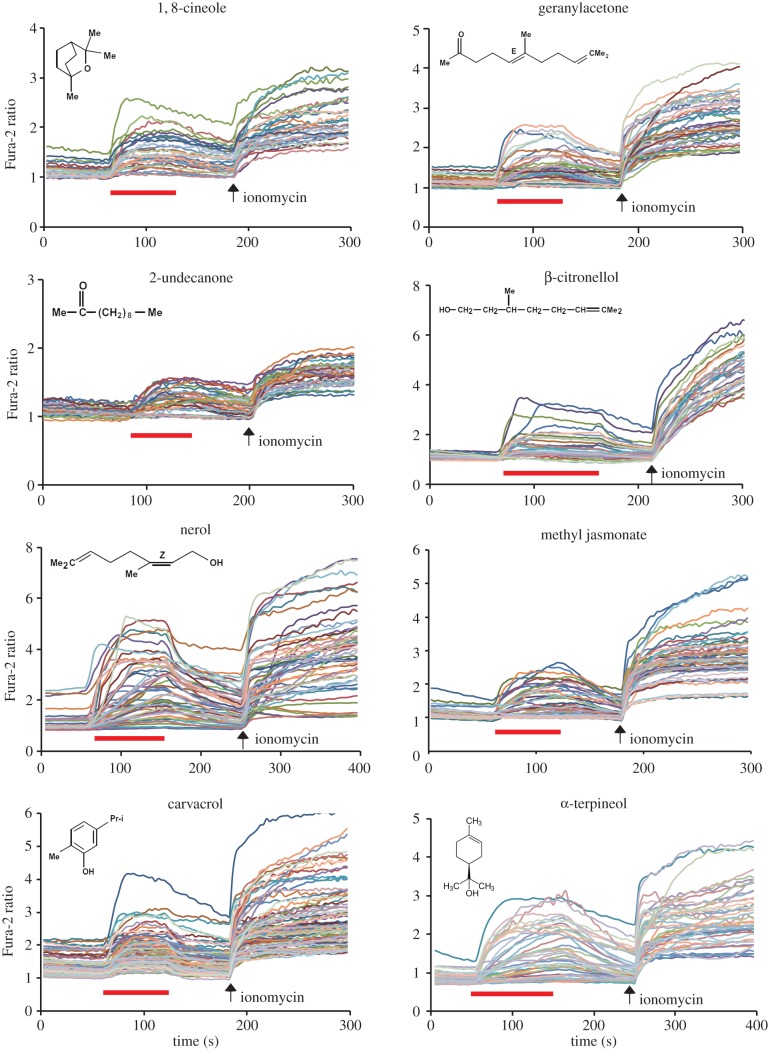
Many plant oil-derived tick repellents activate TmTRPA1b. Activation of TmTRPA1b by eight representative plant oil-derived tick repellents analysed by calcium imaging. Red bars show the period when each compound was added and then washed off. Arrows show the time points when ionomycin was added. The chemical structure of each compound is also shown. The concentration of each compound was 1 mM except for geranylacetone, nerol and carvacrol (0.5 mM).

**Figure 4.**
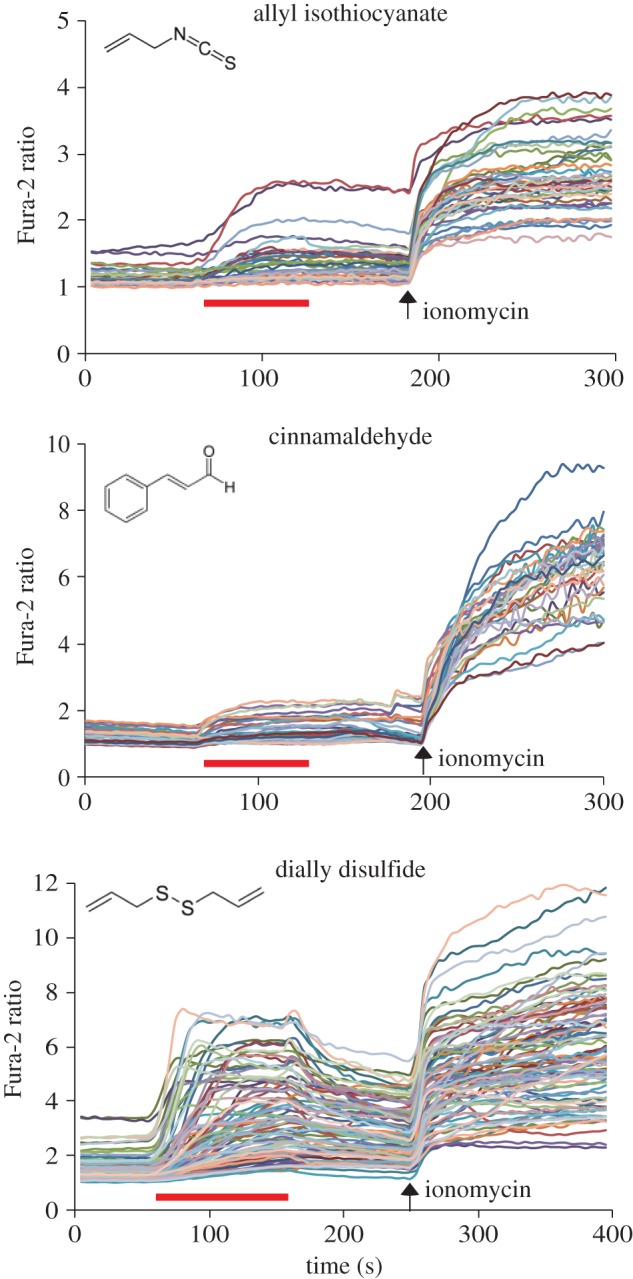
Conserved TmTRPA1b activation by electrophilic compounds. Activation of TmTRPA1b by three electrophiles, allyl isothiocyanate, cinnamaldehyde and dially disulfide, analysed by calcium imaging. Red bars show the period when each compound was added and then washed off. Arrows show the time points when ionomycin was added. Chemical structure of each compound is also shown. The concentration of each compound was 1 mM.

Previous reports have shown that mammalian TRPA1 and DmTRPA1 can be activated by a variety of compounds, including electrophilic compounds, which covalently modify the cysteine residues, and by other compounds (for example, menthol and nifedipine) which do not covalently bind with the channel [5]. Nevertheless, it is not known how far the above chemical activation profiles can be extended to TRPA1 in other species. We therefore tested the activation of three TmTRPA1 isoforms by various chemical compounds and particularly focused on plant-derived compounds with tick-repellent activity [21]. We tested 39 compounds using HEK293 cells expressing TmTRPA1b by calcium-imaging technique and found 27 of them activated it. The list of active and inactive compounds to open TmTRPA1b channel is shown in electronic supplementary material, table S1. We observed robust activation of TmTRPA1b by eight representative plant-derived compounds (1,8-cineole, geranylacetone, 2-undecanone, β-citronellol, nerol, methyl jasmonate, carvacrol and α-terpineol) as shown in figure 3. The activation profiles of TmTRPA1b by other compounds are shown in electronic supplementary material, figure S1*a*,*b*. As 1,8-cineole inhibits human TRPA1 activity [22], its effect on TmTRPA1b appears to be the opposite. The compound 2-undecanone is already used as a major ingredient of commercially available natural arthropod repellents [23]. The chemical structures of TmTRPA1b-activating compounds are diverse (figure 3; electronic supplementary material, figure S1*a*,*b*), suggesting diverse activation mechanisms. These results suggest that at least some of the plant-derived tick/mite repellents activate the TRPA1 channels. Similar to the activation of TRPA1 of other species, such as DmTRPA1, electrophilic compounds such as allyl isothiocyanate (AITC), cinnamaldehyde (CA) and diallyl disulfide also activate TmTRPA1b (figure 4). Thus, the chemical activation profile of TmTRPA1b is the same as that of VdTRPA1 L [12].

**Figure 5.**
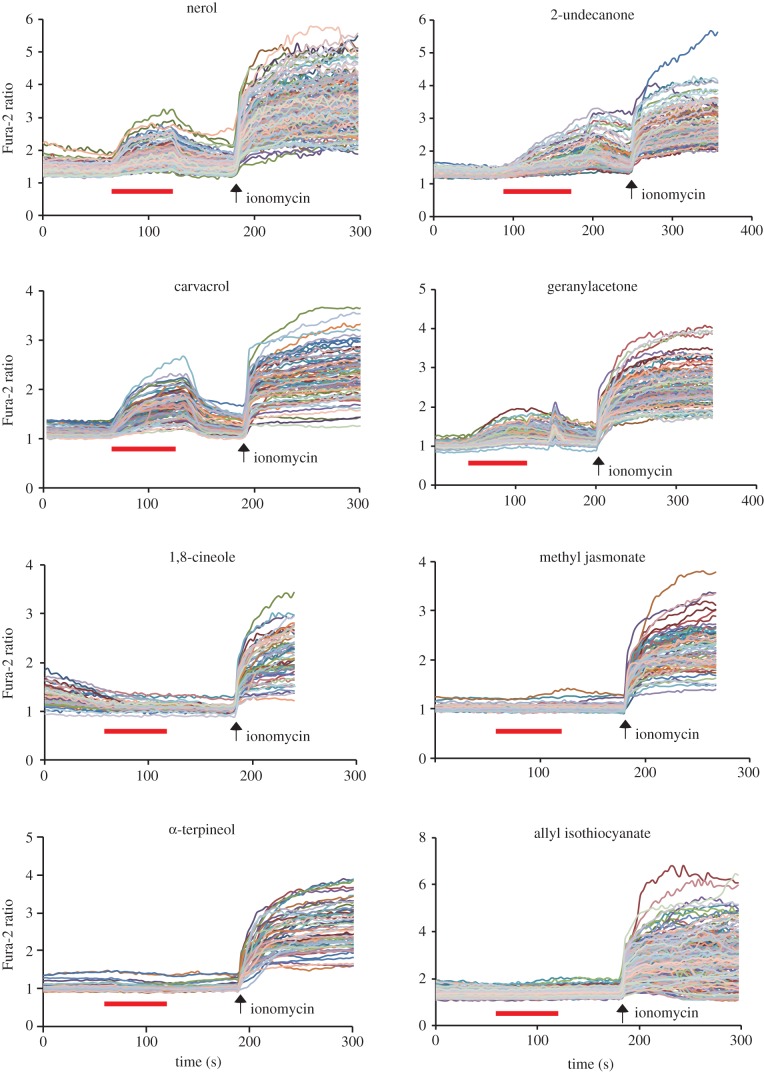
Responses of TmTRPA1a to TmTRPA1b/c activating compounds. Responses of TmTRPA1a to eight representative TmTRPA1b/c activating compounds analysed by calcium imaging. Red bars show the period when each compound was added and then washed off. Arrows show the time points when ionomycin was added. The concentration of each compound was 1 mM except for carvacrol and geranylacetone (0.5 mM).

Although the chemical activation profile of TmTRPA1c was identical to that of TmTRPA1b (electronic supplementary material, table S1 and figure S2*a–d*), we found that only six of the compounds activated TmTRPA1a. These are nerol, 2-undecanone, carvacrol, geranylacetone, eugenol and terpinen-4-ol (figure 5; electronic supplementary material, figure S3). However, many other TmTRPA1b/c-activating compounds such as 1,8-cineole, methyl jasmonate, α-terpineol and AITC failed to activate TmTRPA1a (figure 5; electronic supplementary material, table S1).

### 2.6. Chemical activation of the N-terminal deletion mutants of TmTRPA1b

**Figure 6.**
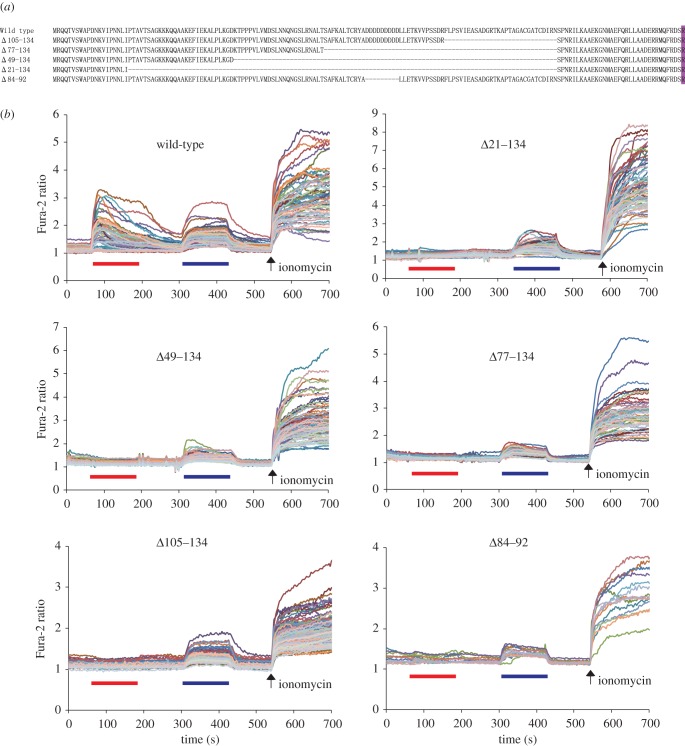
α-terpineol fails to activate five N-terminal deletion mutants of TmTRPA1b. (*a*) The amino acid sequences deleted in five TmTRPA1b mutants (Δ105–134, Δ77–134, Δ49–134, Δ21–134 and Δ84–92) are indicated by dashed lines. The first amino acid of AR1 (R) is marked with purple. (*b*) The traces show the responses of five N-terminal deletion mutants of TmTRPA1b to 1 mM α-terpineol followed by 0.5 mM carvacrol tested by calcium imaging. Red and blue bars show the periods when α-terpineol and carvacrol were added and then washed off, respectively. Arrows show the time points when ionomycin was added.

The above results demonstrate that chemosensitivities of TmTRPA1a and TmTRPA1b isoforms with the unique N-terminal sequences are different. This suggests that the N-terminal sequence is the critical determinant for the chemosensitivity of TmTRPA1 channel. Because TmTRPA1a contains a shorter N-terminal sequence than TmTRPA1b (figure 1*b*), we constructed serial N-terminal deletion mutants (Δ21–134, Δ49–134, Δ77–134 and Δ105–134) as well as Δ84–92 mutant lacking a stretch of nine aspartic acids of TmTRPA1b (figure 6*a*). Protein expression levels and plasma membrane localizations of the five deletion mutants were comparable to those of wild-type by Western blot and immunofluorescence as shown in electronic supplementary material, S5. This indicates that the N-terminal sequence of TmTRPA1b does not significantly affect the protein stability and intracellular localization in HEK293 cells. We then tested the responses of the wild-type and the above five deletion mutants to α-terpineol and carvacrol by calcium imaging, and found that the wild-type was activated by both compounds, but all of the deletion mutants were activated by carvacrol but not α-terpineol (figure 6*b*). These results demonstrate that a stretch of nine aspartic acids (84–92) as well as other amino acids in the N-terminal sequence of TmTRPA1b are necessary for the activation by α-terpineol.

### 2.7. α-terpineol repels *Tropilaelaps mercedesae*

**Figure 7.**
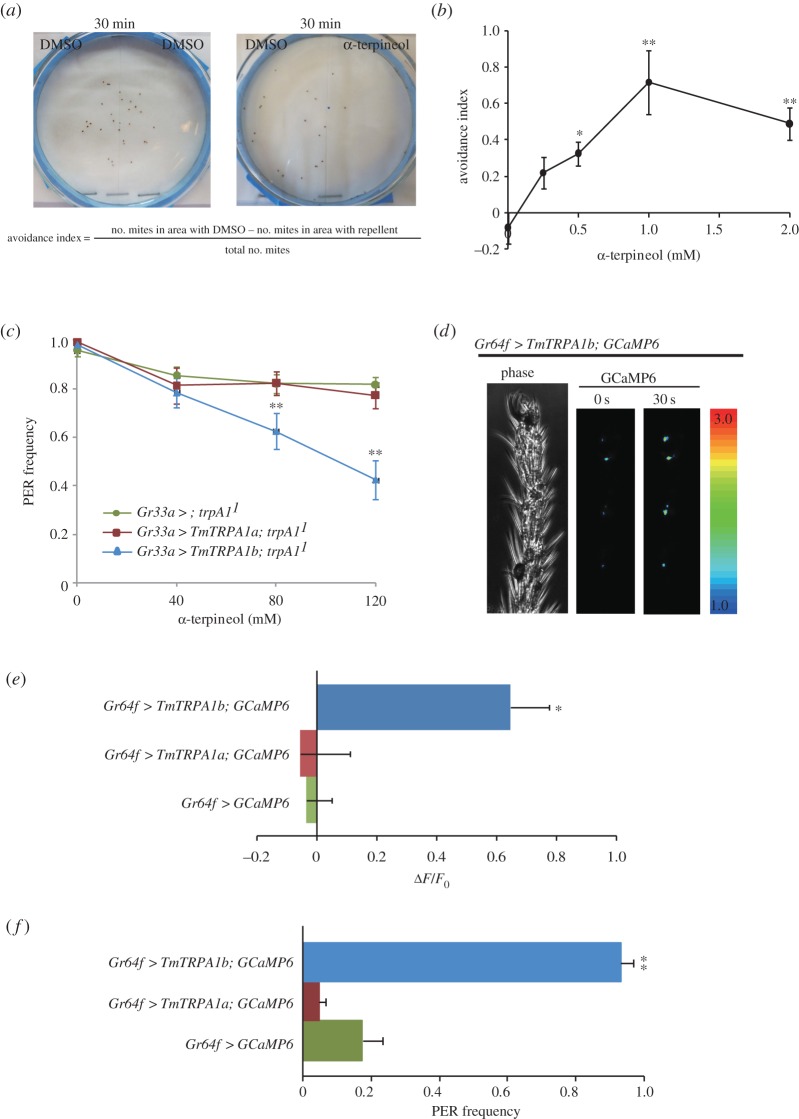
α-terpineol repels *Tropilaelaps mercedesae* and modifies gustatory responses of *Drosophila melanogaster* expressing TmTRPA1b. (*a*) We introduced approximately 20 *Tropilaelaps* mites at the boundary of two filter papers soaked with either α-terpineol or 2% DMSO through nylon mesh, and then the mites were allowed to move for 30 min, after which we counted the number of mites in each area. In the control experiments, both filter papers were soaked with 2% DMSO. We calculated the avoidance index as shown. (*b*) The avoidance index at the different concentrations of α-terpineol. The mean value with error bar (±s.e.m.) is indicated for each concentration. *p*-values (one-way ANOVA followed by the Dunnett post hoc test) at 0.5*, 1** and 2** mM are <0.035, <0.0007 and <0.006, respectively, compared with 0 mM. (*c*) Proboscis extension response (PER) frequency of *trpA1^1^* mutant fruit flies expressing either none (*Gr33a* *>* *; trpA1^1^*), *TmTRPA1b* (*Gr33a* *>* *TmTRPA1b; trpA1^1^*) or *TmTRPA1a* (*Gr33a* *>* *TmTRPA1a; trpA1^1^*) under *Gr33a-Gal4* towards 100 mM sucrose solution containing the different concentrations of α-terpineol. The mean value with error bar (±s.e.m.) is shown for each concentration. Compared to *trpA1^1^*, *p*-values (one-way ANOVA followed by the Dunnett post hoc test) for *trpA1^1^* expressing TmTRPA1b at 80** and 120** mM are <0.017 and <0.00004, respectively. (*d*) Phase and GCaMP6 images of fruit fly expressing both TmTRPA1b and GCaMP6 under *Gr64f-Gal4* (*Gr64f* *>* *TmTRPA1b; GCaMP6*) before (0 s) and at 30 s after applying 10 mM α-terpineol to the distal segments of the foreleg. The increase of GCaMP6 fluorescence (Δ*F*) is indicated by pseudo-colour. (*e*) Intracellular calcium changes (Δ*F*/*F*_0_) of TmTRPA1b and GCaMP6-, TmTRPA1a and GCaMP6- or GCaMP6-expressing sugar taste neuron associated with 5D1 sensilla at 30 s after applying 10 mM α-terpineol. *p*-value (one-way ANOVA followed by the Dunnett post hoc test) of fruit flies expressing both TmTRPA1b and GCaMP6 is less than 0.012 (*) compared with the ones expressing only GCaMP6. (*f*) PER frequency of fruit flies of *Gr64f* *>* *TmTRPA1b; GCaMP6, Gr64f* *>* *TmTRPA1a; GCaMP6* and *Gr64f* *>* *GCaMP6* stimulated by 10 mM α-terpineol. Only fruit flies expressing both TmTRPA1b and GCaMP6 (*Gr64f* *>* *TmTRPA1b; GCaMP6*) (**) show the significant PER (*p* < 0.000002, one-way ANOVA followed by the Dunnett post hoc test compared with the ones expressing only GCaMP6).

Although most of the plant-derived TmTRPA1b-activating compounds also stimulated AmHsTRPA, a honeybee nocisensitive TRPA channel [24], a few compounds such as α-terpineol and carvacrol described above were inactive to open AmHsTRPA channel [12]. Thus, they may strongly repel *Tropilaelaps* mites rather than honeybees. Therefore, we tested the repellent activity of α-terpineol on *Tropilaelaps* mites in the laboratory. We tested the behaviour of *Tropilaelaps* mites placed on two adjacent filter papers soaked with either 2% DMSO or α-terpineol in 2% DMSO covered with a nylon mesh. In the control assay, both filter papers were soaked with 2% DMSO (figure 7*a*). As shown in figure 7*b*, α-terpineol significantly repelled *Tropilaelaps* mites at concentrations of more than 0.25 mM.

### 2.8. Activation of TmTRPA1b is sufficient to induce gustatory avoidance and attraction behaviours in *Drosophila melanogaster*

To test whether the activation of TmTRPA1b by α-terpineol is sufficient to elicit any avoidance behaviour, we characterized the gustatory responses of *D. melanogaster trpA1^1^* mutants [25] expressing TmTRPA1b under *Gr33a-Gal4* to sucrose solution containing α-terpineol by proboscis extension response (PER) [12,24]. *Drosophila melanogaster Gr33a* is one of the gustatory receptor genes widely expressed in aversive taste neurons [26]. The PER frequency of *trpA1^1^* flies did not significantly change even in the presence of 120 mM α-terpineol; however, that of *trpA1^1^* mutants expressing TmTRPA1b but not TmTRPA1a (α-terpineol insensitive isoform, figure 5) was significantly reduced at concentrations more than 80 mM (figure 7*c*). The results demonstrate that in *D. melanogaster*, direct chemical activation of TmTRPA1b could induce gustatory avoidance behaviour when expressed in aversive taste neurons where DmTRPA1 is also present [27].

Similarly, because α-terpineol is a neutral compound for *D. melanogaster*, we tested whether stimulation of TmTRPA1b in the sugar taste neurons by α-terpineol was sufficient to induce gustatory attraction behaviour. We expressed both TmTRPA1b and a calcium-sensor protein, GCaMP6 [28], in the Gr64f-positive sugar taste neurons [29], and GCaMP6 fluorescence was detected in several discrete neurons in the distal segments of foreleg [12]. As shown in figure 7*d*, applying 10 mM α-terpineol to the distal segments of foreleg increased GCaMP6 fluorescence in the Gr64f-expressing sugar taste neurons associated with multiple sensilla including 5D1, 5V1 and 5V2 sensilla [30]. We detected the increase of intracellular calcium level (Δ*F*/*F*_0_) in the sugar taste neuron associated with 5D1 sensilla expressing both TmTRPA1b and GCaMP6, but not expressing either GCaMP6 alone or both TmTRPA1a and GCaMP6 (figure 7*e*). TmTRPA1b stimulation by α-terpineol was able to activate the sugar taste neurons, and as a result, the application of 10 mM α-terpineol to the forelegs was sufficient to induce significant PER in the fruit flies expressing both TmTRPA1b and GCaMP6, but not either GCaMP6 alone or both TmTRPA1a and GCaMP6 (figure 7*f*). The results demonstrate that manipulating the activity of taste neurons by the introduction of TmTRPA1b and by the application of α-terpineol is sufficient to modify the gustatory behaviours of fruit flies.

### 2.9. Application of α-terpineol to *Tropilaelaps mercedesae*-infested colonies

For reproduction of *T. mercedesae*, the female mite needs to enter into brood cell containing the fifth instar larvae prior to capping (reproductive phase) [16]. Subsequently, the mother mite starts to lay eggs within the sealed brood cell [16,17]. As α-terpineol is capable of repelling *Tropilaelaps* mites as shown above, we tested whether it could repress female mites from entering brood cells containing the fifth instar larvae. Brood cells treated with α-terpineol (1 µl of 0.3 M solution) showed 29.5% significantly lesser infestation than that in the control cells treated using DMSO (infestation in control cells = 47.9%, infestation in cells treated with α-terpineol = 33.8%; *p* < 0.018, Mantel–Haenszel test). The results demonstrate that the appropriate concentration of α-terpineol could repress the infestation of *T. mercedesae* females to brood cells with the fifth instar worker larvae in the colony.

## 3. Discussion

### 3.1. *Tropilaelaps mercedesae* expresses three TmTRPA1 isoforms

*Tropilaelaps mercedesae* expresses three *TmTRPA1* mRNA isoforms, *TmTRPA1a, TmTRPA1b* and *TmTRPA1c*, by transcription from the different initiation sites (figure 1*a*). All isoforms contain the same number of ARs but unique N-terminal amino acid sequences. Similar isoforms are also present for *DmTRPA1*, such as *TrpA1-RG* and *TrpA1-RI* (FlyBase, http://flybase.org). Although DmTRPA1 isoform with short N-terminal sequence is sensitive to both heat and electrophile stimulations, the isoform with long N-terminal sequence is only activated by chemical stimulation and is preferentially expressed in the chemosensory neurons. Thus, this could be a mechanism to discriminate heat and chemical stimuli by using a single *TRPA1* gene in *D. melanogaster* [31,32]. Similarly, in *V. destructor*, two TRPA1 isoforms (VdTRPA1 L and VdTRPA1S) with the unique N-terminal sequences and different numbers of ARs are present. In contrast with VdTRPA1 L, VdTRPA1S containing fewer ARs is not activated by chemical stimulation when expressed in HEK293 cells and *D. melanogaster* [12]*.* Only VdTRPA1 L appears to be a direct sensor involved in chemo-reception with its exclusive expression in the front legs of *V. destructor*. VdTRPA1S was proposed to be a downstream component of signalling pathways activated by certain sensory stimuli [12]. Three TmTRPA1 isoforms are different from the above DmTRPA1 and VdTRPA1 isoforms because all of them can be activated by heat (figure 2) with the same number of ARs. However, as shown in figures 3–5, and electronic supplementary material, table S1 and figures S1–S3, TmTRPA1a and TmTRPA1b/c have the different chemical sensitivities for activation. The long TmTRPA1b/c is activated by more compounds than the short TmTRPA1a. These results demonstrate that the N-terminal amino acid sequence of TmTRPA1 facing the cytosol is the critical determinant for the chemical activation. In fact, partially deleting N-terminal amino acid sequence of TmTRPA1b resulted in the loss of reactivity to α-terpineol but not carvacrol (figure 6). Both nine aspartic acids (84–92) and 30 amino acids (105–134) were independently necessary for TmTRPA1b activation by α-terpineol (figure 6), suggesting that many amino acids in the N-terminus would be required. Alternatively, whole N-terminus of TmTRPA1b may operate as the functional module to support the chemical reactivity. Amino acid substitutions in the N-terminal amino acid sequence of TmTRPA1b by random mutagenesis followed by testing the activities of mutant channels may help to delineate the specific amino acids responsible for the chemical activation. Furthermore, this is the first example to show the evolutionary plasticity of TRP channels by mutations in the *cis*-regulatory elements (changing the transcriptional initiation sites) to generate the isoforms with different chemical sensitivities.

TmTRPA1c is exclusively present in the body but not in any legs of the *Tropilaelaps* mite by our method, suggesting that it may be specifically expressed in the brain and/or internal organs of body (figure 1*c,d*). TmTRPA1a and TmTRPA1b are present throughout the whole body but at a higher level in the body and the first legs, respectively (figure 1*c,d*). This may suggest that TmTRPA1a and TmTRPA1b are expressed in the different types of sensory neurons enriched in the body and the first legs, respectively. It is difficult to predict how many *TmTRPA1* mRNA isoforms are present from the genomic sequence. The deep transcriptome sequencing may help to identify all isoforms present in the *Tropilaelaps* mite. The first legs of the *Tropilaelaps* mite morphologically differentiate from the other legs (figure 1*c*) and are always held upright, demonstrating that they function as sensory organs like insect antennae [20]. TmTRPA1b is likely to be a direct sensor involved in heat- and chemo-reception by the major sensory organ (the front legs) of the *Tropilaelaps* mite.

### 3.2. Physiological roles of chemosensitive TmTRPA1 for *Tropilaelaps mercedesae*

We found that TmTRPA1 is activated by a variety of plant-derived compounds including electrophiles (figures 3–5; electronic supplementary material, table S1 and figures S1–S3). Many active compounds appear to contain either a long carbon chain or ring structure, suggesting that they may affect the integrity and/or fluidity of plasma membrane. It could indirectly activate TmTRPA1 channels; however, the lack of activation by octanoic acid, decanoic acid and linoleic acid (electronic supplementary material, table S1) suggests that this is unlikely to occur. These relatively hydrophobic TmTRPA1-activating compounds may directly interact with the transmembrane segments of channel to open the gates. As honeybees collect nectar, pollen and resin from various plant species and bring them to the hive, *Tropilaelaps* mites would have chances to be exposed to the above TmTRPA1-activating compounds, which are potentially hazardous. Some components of plant essential oils are neurotoxic to insects and affect the activities of biogenic amine (tyramine and octopamine) receptors [33], GABA_A_ receptor ion channels [34] and acetylcholine esterase [35], suggesting that they could have similar neurotoxic effects on *Tropilaelaps* mites. Thus, *Tropilaelaps* mites, using TmTRPA1, may avoid these compounds, as shown in figure 7*a,b*. We do not have direct evidence to support this hypothesis because the mock-treated mites for RNAi-knockdown are incapable of moving; however, replacing DmTRPA1 with TmTRPA1b in *D. melanogaster* was sufficient to induce gustatory avoidance to TmTRPA1b (but not DmTRPA1) activating compound, α-terpineol (figure 7*c*). This demonstrates that chemical activation of TmTRPA1b in aversive taste neurons is sufficient to induce avoidance behaviours by the activating compounds. Meanwhile, the activation of TmTRPA1b in the sugar taste neurons by α-terpineol could induce PER (figure 7*f*), showing that the behavioural outcome of TmTRPA1b activation is context-dependent in *D. melanogaster*.

### 3.3. Potential use of TmTRPA1-activating compounds to control both *Tropilaelaps mercedesae* and *Varroa destructor* in apiculture industry

In many Asian counties, managed *A. mellifera* colonies are often infested by both *Varroa* and *Tropilaelaps* mites. Thus, miticides and other chemical compounds such as sulfur, formic acid and thymol have been used to control both mite species [16]. However, the efficacy of the above treatments would be different between two mite species, and *Tropilaelaps* mites may also develop resistance to pyrethrids (tau-fluvalinate and flumethrin) due to the mutations in the voltage-gated Na^+^ channel as demonstrated in *Varroa* mites [36,37]. Thus, developing effective control strategies for both mite species is crucial for the apiculture industry worldwide.

Given the repellent activity of α-terpineol towards *Tropilaelaps* mites in a laboratory assay (figure 7*a,b*), we found that applying 0.3 M α-terpineol into brood cells of hives with fifth instar larvae repelled female mites from entering. However, α-terpineol failed to repel the mites at 0.1 M, suggesting that the activation of TmTRPA1 by α-terpineol may become ineffective inside a honeybee hive where lots of other odours and tastants are present at high concentrations. Because α-terpineol was also effective for *Varroa* mites by the same assay [12], it can be used as the potential compound to control both *Tropilaelaps* and *Varroa* mites. Although α-terpineol did not activate AmHsTRPA [12], its short-term effects on honeybees and its long-term effects on the entire colony are yet to be characterized. Nevertheless, the appropriate use of TmTRPA1-activating natural compounds derived from plants may serve as an alternative method to control *Tropilaelaps* mites in the apiculture industry.

### 3.4. Functional conservation of TRPA1 channels between two major species of honeybee ectoparasitic mite

TmTRPA1 is the sixth arthropod TRPA1 to be characterized in detail besides DmTRPA1, *Anopheles gambiae* TRPA1, *B. mori* TRPA1, *Helicoverpa armigera* TRPA1 and VdTRPA1. It is the second TRPA1 characterized in Acari, which includes mites and ticks, representing the majority of ectoparasites of various animals and plants. As we previously reported, VdTRPA1 and DmTRPA1 share some channel properties (for example, electrophile sensitivity); however, they are activated by the different compounds [12]. This is consistent with the species-dependent temperature/chemical activation of vertebrate TRPA1 channels [38–41]. These results led us to hypothesize that amino acid substitutions in TRPA1 would be often driven by adaptive evolution to adjust the physiological functions in association with different specific habitats and life histories of the respective animal species [42]. Amino acid sequences of TmTRPA1b and VdTRPA1 L share 87% identity and 92% similarity, and there are no differences in their channel properties as far as we tested. Both are activated by the same compounds. This is intriguing because the minimal amino acid substitutions are sufficient to change the properties of TRPA1 channel, such as the activation temperature directionality [43]. The important roles of TRPA1 to mediate honeybee–mite interactions as well as to adapt to the hive environment may keep the channel properties of VdTRPA1 and TmTRPA1 similar to each other. In fact, we found six positively selected amino acids in the ancestor of VdTRPA1 and TmTRPA1. These are 239S, 334I, 435N, 523Y, 677N and 1133R (the numbers refer to the positions in TmTRPA1b), and the first five amino acids are located in the AR3, AR5, AR8, AR10 and AR14 of TmTRPA1b, respectively. This may suggest the importance of ARs to modulate the gating property of the TRPA1 channel. Nevertheless, as shown in figures 1 and 3–5, generation of the isoforms and their expression profiles appear to be different and generate the diversity of TRPA1 channels in two species of honeybee ectoparasitic mite. Very little is known about the sensory physiology of *T. mercedesae* despite its critical roles in host finding and reproduction, and thus, further study on the TRP channels would aid in developing novel control methods to fight this major pest of the apiculture industry in Asia.

## 4. Material and methods

### 4.1. 5′ and 3′RACE of *TmTRPA1*

Based on VdTRPA1 and TRPA1 sequences of two mite/tick species, *M. occidentalis* (XM_003748080.1) and *I. scapularis* (XM_002405489.1), we first designed degenerate primers (5′-YGARGCKGCSAARAAYGCKTCSGCYAACGC-3′ and 5′-GAACATGSCVGCGCARTGSARTGGCGTCAT-3′ for the first PCR; 5′-ACVGAKCGRCCYTCYTTRTCYGTDGC-3′ and 5′-GAACATGSCVGCGCARTGSARTGGCGTCAT-3′ for the second PCR) to amplify a partial *TmTRPA1* cDNA at 5′ end using *T. mercedesae* total RNA by nested RT-PCR. The sequence of amplified band was highly similar to that of TRPA1 from other species. We then designed the primer sets for 5′ and 3′ RACE using this cDNA fragment. *Tropilaelaps mercedesae* total RNA and two primers, 5′-CGCGGATCCACAGCCTACTGATGATCAGTCGATG-3′ (for the first PCR) and 5′-TCGCAGAACTCGAGCAGAGCCCTCA-3′ (for the second PCR), were used for 5′RACE with SMARTer RACE cDNA Amplification Kit (Clontech Laboratories). 3′RACE was carried out as above except the following two primers were used: 5′-GTCCACTCGCATCTACTCGACCAG-3′ (for the first PCR) and 5′-AAGAACCGCTCGAGTACCGCAGTG-3′ (for the second PCR). To fully extend the 5′ end of TmTRPA1 cDNA, two additional primers, 5′-ACGTTCGTCAGCTGCCAGTAACCGTTG-3′ (for the first PCR) and 5′-ACTCTGCCATATTGCCCTTCACTCCGCAG-3′ (for the second PCR), were used with 5′-Full RACE Kit (TAKARA).

### 4.2. Construction of TmTRPA1-expressing vector for mammalian cells

We isolated full-length *TmTRPA1a*, *TmTRPA1b* and *TmTRPAc* cDNAs by nested RT-PCR with *T. mercedesae* total RNA and two primers for the first PCR, either 5′-AAAATCGTCGAAGGCTACTGCCTC-3′ (for *TmTRPA1a*), 5′-AATCCGCGTTCTACCCTTGACCGT-3′ (for *TmTRPA1b*) or 5′-GAGCAGATCTCCATCATCCAGTCG-3′ (for *TmTRPA1c*) and 5′-GTCCGAAGCCACCAAGGACGTAATAGG-3′. The second PCR was then carried out using the first PCR product as a template and the following two primers: either 5′-AATTTGCGGCCGCACC**ATG**GCGCGAAGGCTCATGAGAGATGCTTC-3′ (for *TmTRPA1a*), 5′-AATTTGCGGCCGCACC**ATG**CGTCAACAAACCGTGTCTTGG-3′ (for *TmTRPA1b*) or 5′-AATTTGCGGCCGCACC**ATG**GACCACGGAACGGTTCACACG-3′ (for *TmTRPA1c*) and 5′-TTTCTAGACTCTTGACATCCGTTTGTCCATCTAGCTGTTC-3′. The second PCR products were digested with Not I and Xba I, and then cloned in pAc5.1/V5-His B vector (Life Technologies) in which *D. melanogaster actin 5C* promoter was replaced with CMV promoter. The TmTRPA1 protein expressed by this construct was tagged with a V5-epitope at the C-terminus and this was used for verifying the expression and the cellular localization in HEK 293 cells by Western blot and immunofluorescence with rabbit anti-V5-epitope antibody (Sigma-Aldrich), respectively. The staining patterns of V5-epitope-tagged TmTRPA1 channels were compared with FITC-WGA, which specifically labels the plasma membrane [44]. A construct expressing untagged TmTRPA1 protein was then prepared using the above DNA construct as a template, the above primer with the initiation codon and the primer 5′-TTTCTAGACTCTACTTGACATCCGTTTGTCCATC-3′. This DNA construct was used for all of the experiments described in the text. The TmTRPA1b deletion mutants were generated by overlap extension PCR [45].

### 4.3. RT-PCR

Total RNA was isolated from the *Tropilaelaps* mite's first legs, second–fourth legs and whole bodies without legs and mouth parts using Trizol reagent (Life Technologies). In total, 0.1 µg of total RNA was used for the reverse transcription reaction using random primer and ReverTra Ace reverse transcriptase (TOYOBO). The RT products were then used for the first PCR with the following primers: either 5′-GCTGATGTCCCTCGCAACTGTGTT-3′ (for *TmTRPA1a*), 5′-TGGCAGAAGGAAAAGCCCGTAGGA-3′ (for *TmTRPA1b*) or 5′-GCCTGACTGACTGCATGAGAAGTC-3′ (for *TmTRPA1c*) and 5′- CGAAACTGCATCCGACGTTCGTCA-3′. The second PCR was then carried out using the first PCR products as templates and two primers, either 5′-AAAATCGTCGAAGGCTACTGCCTC-3′ (for *TmTRPA1a*), 5′-AATCCGCGTTCTACCCTTGACCGT-3′ (for *TmTRPA1b*) or 5′-GAGCAGATCTCCATCATCCAGTCG-3′ (for *TmTRPA1c*) and 5′- CCAGTAACCGTTGAAACTCTGCCA-3′. The resulting PCR products were sequenced to verify their identities.

### 4.4. Ca^2+^-imaging method (HEK293 cells)

For Ca^2+^-imaging, 1 µg of TmTRPA1 expression vector and 0.1 µg of pCMV-DsRed expression vector were transfected to HEK293 cells in OPTI-MEM medium (Life Technologies) using Lipofectamine Plus reagents (Life Technologies). After incubating for 3–4 h at 37°C, cells were reseeded on cover glasses and further incubated at 33°C. The cells were used for the experiments at 20–40 h after transfection. Transfected HEK293 cells on a cover glass were incubated in culture media containing 5 µM Fura-2 AM (Life Technologies) at 33°C for 1–2 h. The cover glass was washed and Fura-2 fluorescence was measured in a standard bath solution containing (in mM) 140 NaCl, 5 KCl, 2 MgCl_2_, 2 CaCl_2_, 10 HEPES and 10 glucose and pH 7.4 adjusted with NaOH. Calcium chloride was omitted and 5 mM EGTA was added in the calcium-free bath solution. A cover glass was mounted in a chamber (RC-26G, Warner Instruments Inc.) connected to a gravity flow system to deliver hot bath solution and bath solution containing various compounds. The concentration of each compound was 1 mM except for carvacrol, geranylacetone, nerol (0.5 mM), menthol (3 mM) and creosote (0.1%). The emitted fluorescence (510 nm) by 340 and 380 nm were measured by a CCD camera (CoolSNAP ES, Roper scientific photometrics). Data were acquired and analysed by IPlab software (Scanalytics Inc.).

### 4.5. Genetics

*UAS-TmTRPA1a, UAS-TmTRPA1b* and *UAS-TmTRPA1c* fruit flies were generated by integrating the transgenes to 68A4 by PhiC31 integrase-mediated recombination event [46]. These transgenes were driven by *Gr33a-Gal4* [26] under *trpA1^1^* background [25]. Each of the above transgenes was recombined with *20XUAS-IVS-GCaMP6 s* [28] on the third chromosome, and then their expression was driven by *Gr64f-Gal4* [29] with two copies for each *UAS* and *Gal4* transgene.

### 4.6. PER assay of *Drosophila melanogaster*

PER assay was basically performed as described in [12]. Four- to five-day-old fruit flies were starved overnight on wet Kim Wipe, anaesthetized on ice and affixed to a tooth pick. Fruit flies recovered in a humidified chamber for at least 2 h at room temperature before testing. Each subject was checked for intact PER before beginning the experiments. Flies that did not show the reflex were discarded. During the PER assay, the fruit fly was first satiated with water, then 100 mM sucrose solution containing none or the different concentrations of α-terpineol (WAKO) shown in figure 6*c* was touched to the forelegs with a pipette tip. If the proboscis was extended and contact with the sucrose solution was maintained for 3 s, the response was scored as 1. If the contact of proboscis was brief, a 0.5 was awarded. If the proboscis failed to contact the solution within 5 s of offering, a 0 was awarded. Each fruit fly was offered compounds seven times per experiment and between offering water was given to satiation. If α-terpineol was accepted on first offering, PER frequency was calculated for the second through to the seventh offerings (sum of six scores per fruit fly divided by six). Three groups of 7–8 flies per genotype were tested. For PER induction by α-terpineol, the forelegs of fruit flies prepared as above were touched with the 10 mM solution and then the extension of proboscis was scored followed by checking the intact PER by sucrose.

### 4.7. GCaMP6 imaging in *Drosophila melanogaster*

The female forelegs for GCaMP6 imaging were prepared as described previously [30], except that the distal segments were covered with 5 µl of water on a glass slide. Three to four images were obtained first (0 s), and then time-lapse recording (every 0.5 s for 30 s) by a CCD camera (RETIGA 2000-RV, Roper Scientific Photometrics) was started by adding 5 µl of 20 mM α-terpineol. Data were acquired and analysed by Image-Pro Plus software (Media Cybernetics, Inc.).

### 4.8. Repellent assay with *Tropilaelaps mercedesae* in a laboratory

Two filter papers were soaked with either 2% DMSO or repellent in 2% DMSO and then covered with nylon mesh after joining them side by side. *Tropilaelaps* mites (approx. 20) were aligned at the boundary of two filter papers at the beginning of assay (0 min) and then allowed to move for 30 min in the testing arena (9 cm diameter circle). The number of mites in the area with either DMSO or repellent was counted after 30 min and the avoidance index was calculated. Two filter papers soaked with 2% DMSO were used as a control assay. The experiments were repeated three times for each concentration of α-terpineol.

### 4.9. Bioassay of α-terpineol with *Tropilaelaps mercedesae*-infested *Apis mellifera* colonies

One μl of 0.3 M α-terpineol was applied to each worker brood cell containing the fifth instar larvae in *Tropilaelaps* mite-infested honeybee colonies. An equal number of cells was also treated with 1 µl of DMSO and used as a control. After 2 days, the sealed cells were opened and inspected, and the number of mite-infested cells was counted. The experiment was repeated four times with the different colonies and 172 brood cells in total were tested for each experimental group.

## Supplementary Material

Supplementary figures and legends, Table S1

## Data accessibility

The accession numbers for TmTRPA1a, TmTRPA1b and TmTRPA1c are LC126329, LC126328 and LC126327, respectively.
