# Supplementary material for: Isoform-specific modulation of the chemical sensitivity of conserved TRPA1 channel in the major honeybee ectoparasitic mite, Tropilaelaps mercedesae
Source: Open Biol. 2016 Jun 15;6(6):160042. doi: 10.1098/rsob.160042 (PMC4929936; doi:10.1098/rsob.160042)

Table S1 List of active and inactive compounds to open TmTRPA1 channels

Active compounds to open TmTRPA1a	Inactive compounds to open TmTRPA1a	Active compounds to open TmTRPA1b/c	Inactive compounds to open TmTRPA1b/c
Eugenol	Cinnamaldehyde	Eugenol	Camphor
Carvacrol (0.5 mM)	1,8-Cineole	Cinnamaldehyde	o-Methoxyphenol
2-Undecanone	AITC	1,8-Cineole	2-Methoxy-4-methylphenol
Geranylacetone (0.5mM)	Creosote (0.1%)	AITC	Linoleic acid
Terpinen-4-ol	Methyl jasmonate	Carvacrol (0.5mM)	N,N-Diethyl-2-phenylacetamide
Nerol (1mM)	2-Dodecanone	Creosote (0.1%)	Octanoic acid
	α-Terpineol	Methyl jasmonate	Decanoic acid
	Diallyl disulfide	2-Undecanone	1-Octanal
	3,7-Dimethyl-6-octenal	Nerolidol	Borneol
	2-Ethyl-1,3-hexanediol	Thujone	Coumarin
	Geraniol	2-Dodecanone	Methyl salicylate
	Carveol	Geranylacetone (0.5 mM)	Verbenone
	Lauric acid	Myrtenal	
	1-Octanol	Terpinen-4-ol	
	Nerolidol	β-Citronellol	
	Thujone	α-Terpineol	
	Myrtenal	Nerol (0.5 mM)	
	β-Citronellol	Diallyl disulfide	
	β-cyclocitral	3,7-Dimethyl-6-octenal	
	Menthol (3 mM)	2-Ethyl-1,3-hexanediol	
	Thymol	β-cyclocitral	
	Camphor	Menthol (3 mM)	
	o-Methoxyphenol	Geraniol	
	2-Methoxy-4-methylphenol	Carveol	
	Linoleic acid	Thymol	
	N,N-Diethyl-2-phenylacetamide	Lauric acid	
	Octanoic acid	1-Octanol	
	Decanoic acid		
	1-Octanal		
	Borneol		
	Coumarin		
	Methyl salicylate		
	Verbenone		

All compounds were tested at 1mM concentration except carvacrol, creosote, geranylacetone, nerol, and menthol. The concentrations used are indicated with parentheses.

Supplementary figure legends

Supplementary figures 1a and 1b

Activation of TmTRPA1b by 16 plant-derived compounds analyzed by calcium imaging. Red bars show the period when each compound was added, and then washed off. Arrows indicate the time points when we added ionomycin. Chemical structure of each compound is also shown except creosote which is a mixture of different compounds. The concentration of each compound was 1 mM except for creosote (0.1%), and menthol (3mM).

Supplementary figures 2a-2d

Activation of TmTRPA1c by 27 plant-derived compounds analyzed by calcium imaging. Red bars show the period when each compound was added, and then washed off. Arrows indicate the time points when we added ionomycin. The concentration of each compound was 1 mM except for creosote (0.1%), geranylacetone (0.5mM), carvacrol (0.5 mM), nerol (0.5mM), and menthol (3mM).

Supplementary figure 3

Activation of TmTRPA1a by eugenol and terpinen-4-ol analyzed by calcium imaging. Red bars show the period when each compound was added, and then washed off. Arrows indicate the time points when we added ionomycin. The concentration of each compound was 1 mM.

Supplementary figure 4

(A) Proteins expressed in HEK293 cells transfected with empty vector (Mock), TmTRPA1b wild type-, and the five deletion mutants (Δ84-92, Δ105-134, Δ77-134, Δ49-134, and Δ21-134)-expressing constructs were analyzed by western blot. The size (kD) of protein molecular weight marker (MW) is at the left. (B) Localizations of plasma membrane-bound FITC-WGA and either TmTRPA1b wild type, or the five deletion mutant (Δ84-92, Δ105-134, Δ77-134, Δ49-134, or Δ21-134) tagged with V5-epitope in the transfected HEK293 cells by immunofluorescence. The merged images are also shown.

Supplementary figure 1a

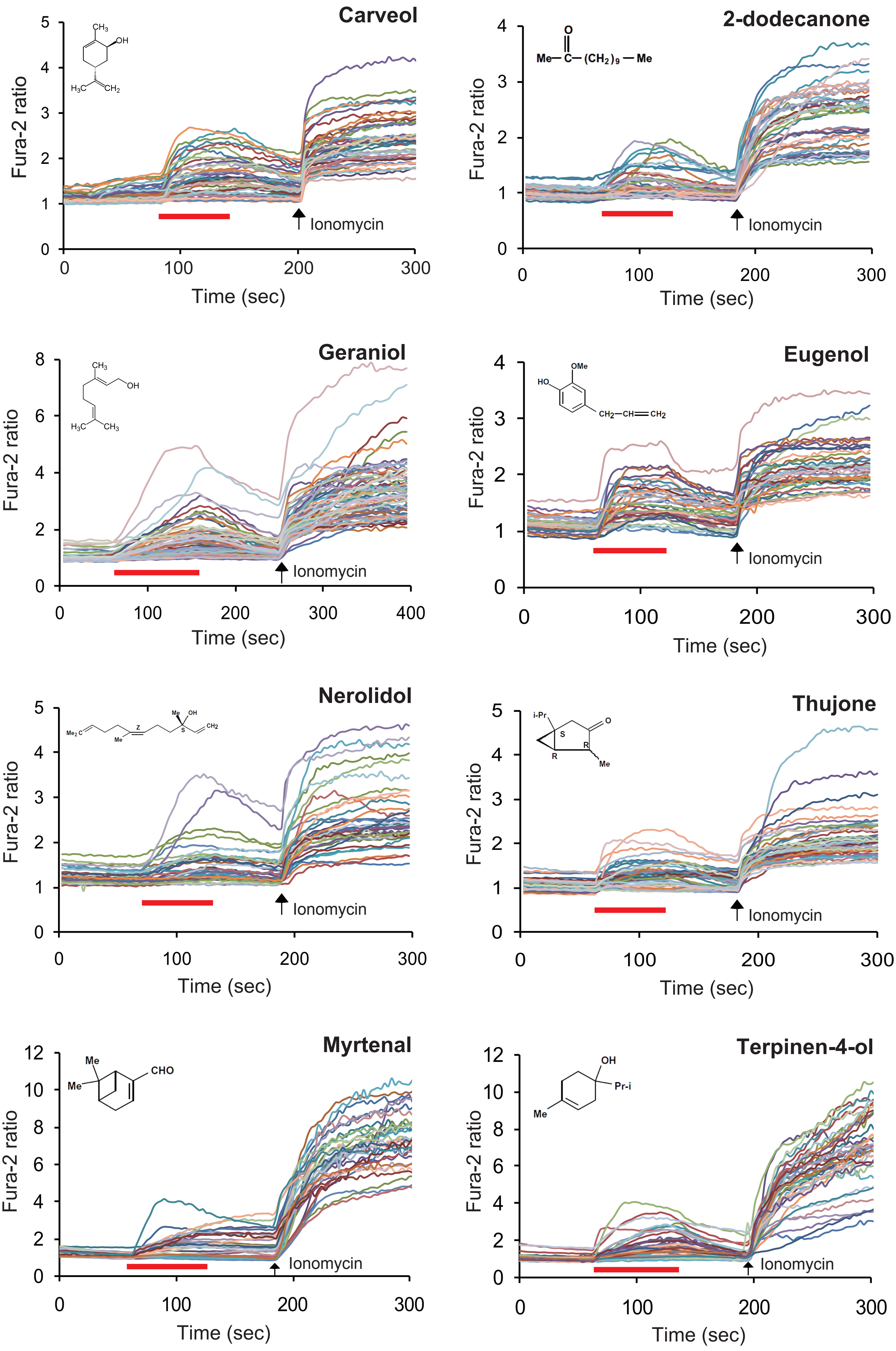

Supplementary figure 1b

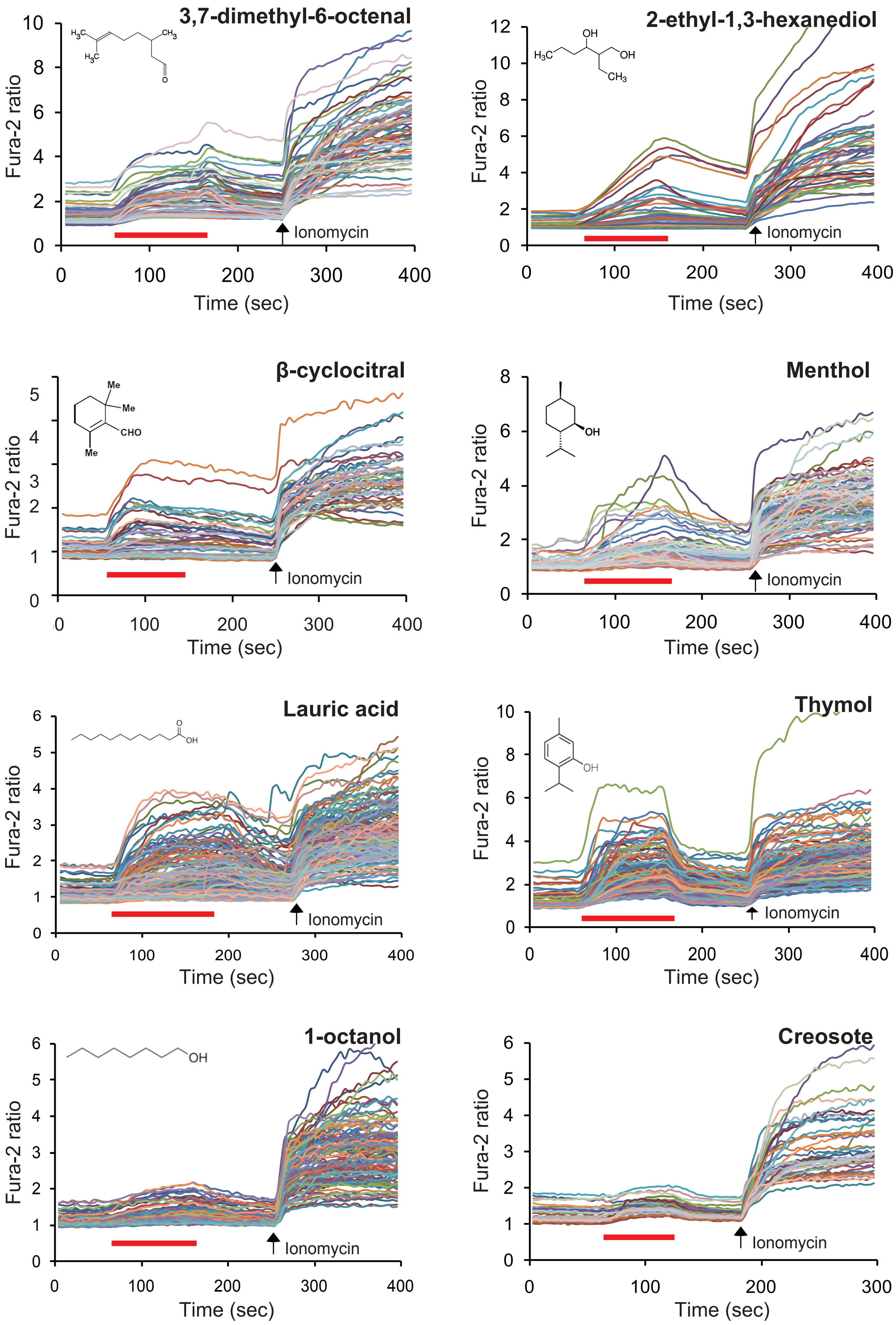

Supplementary figure 2a

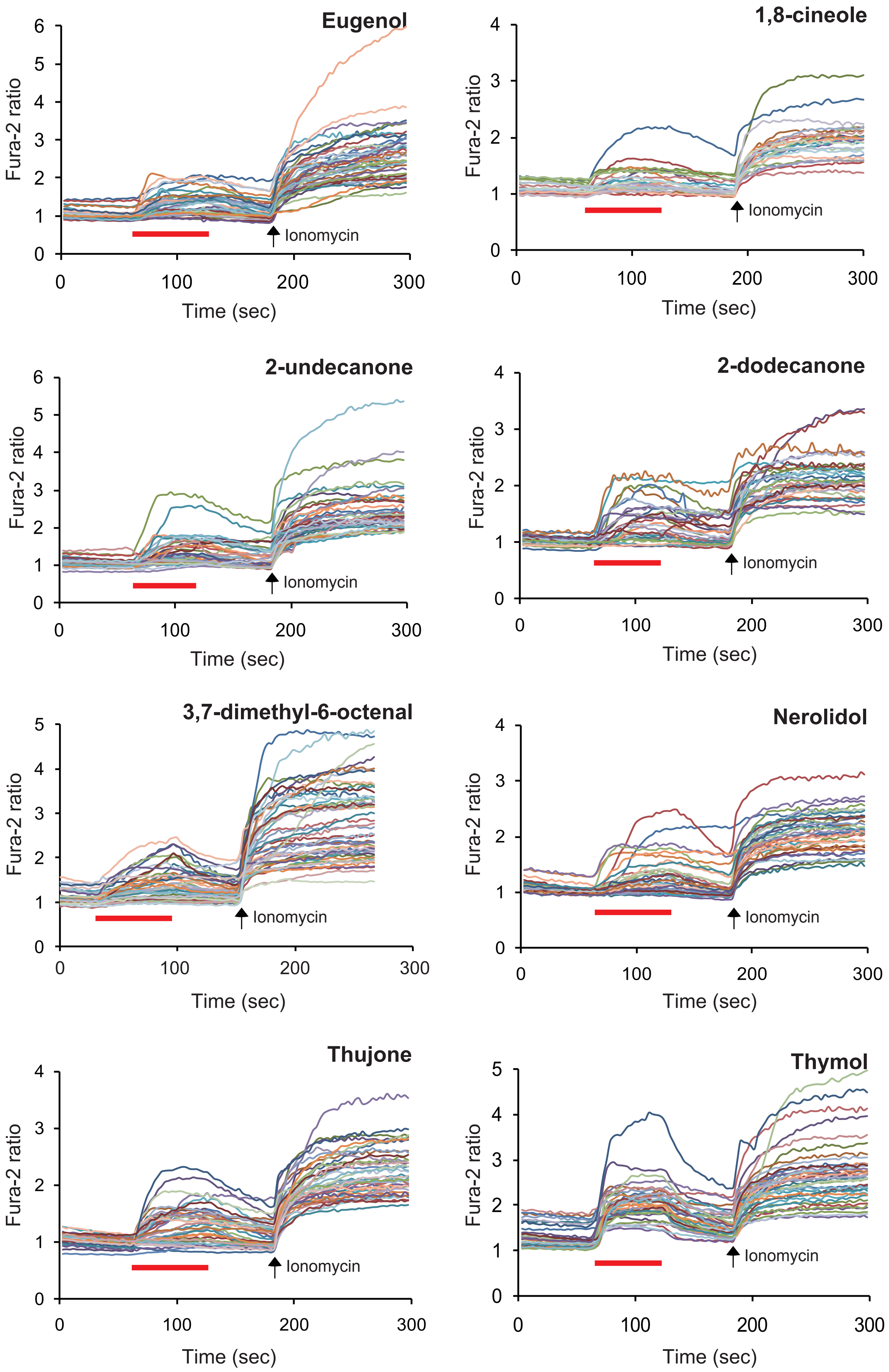

Supplementary figure 2b

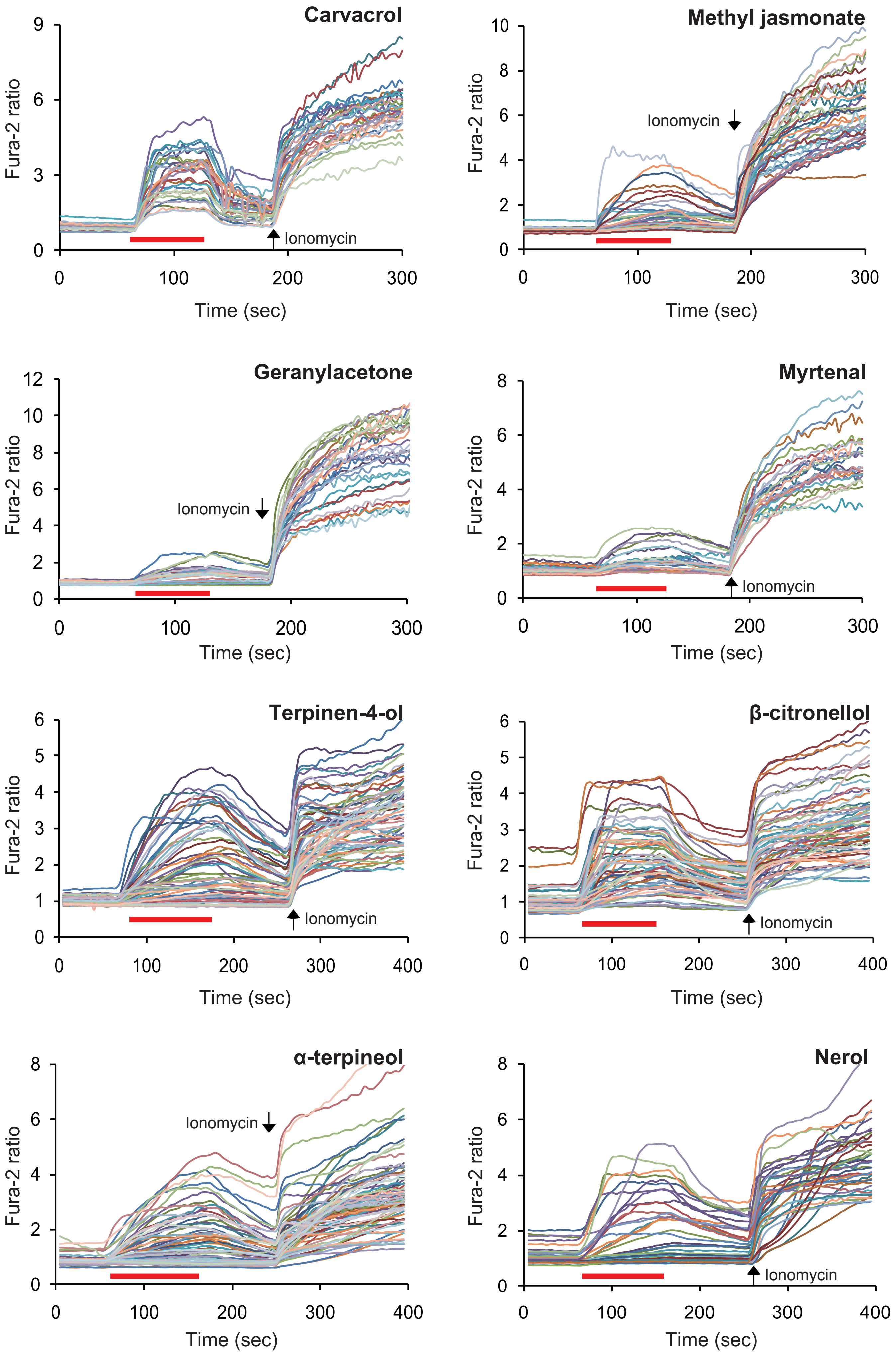

Supplementary figure 2c

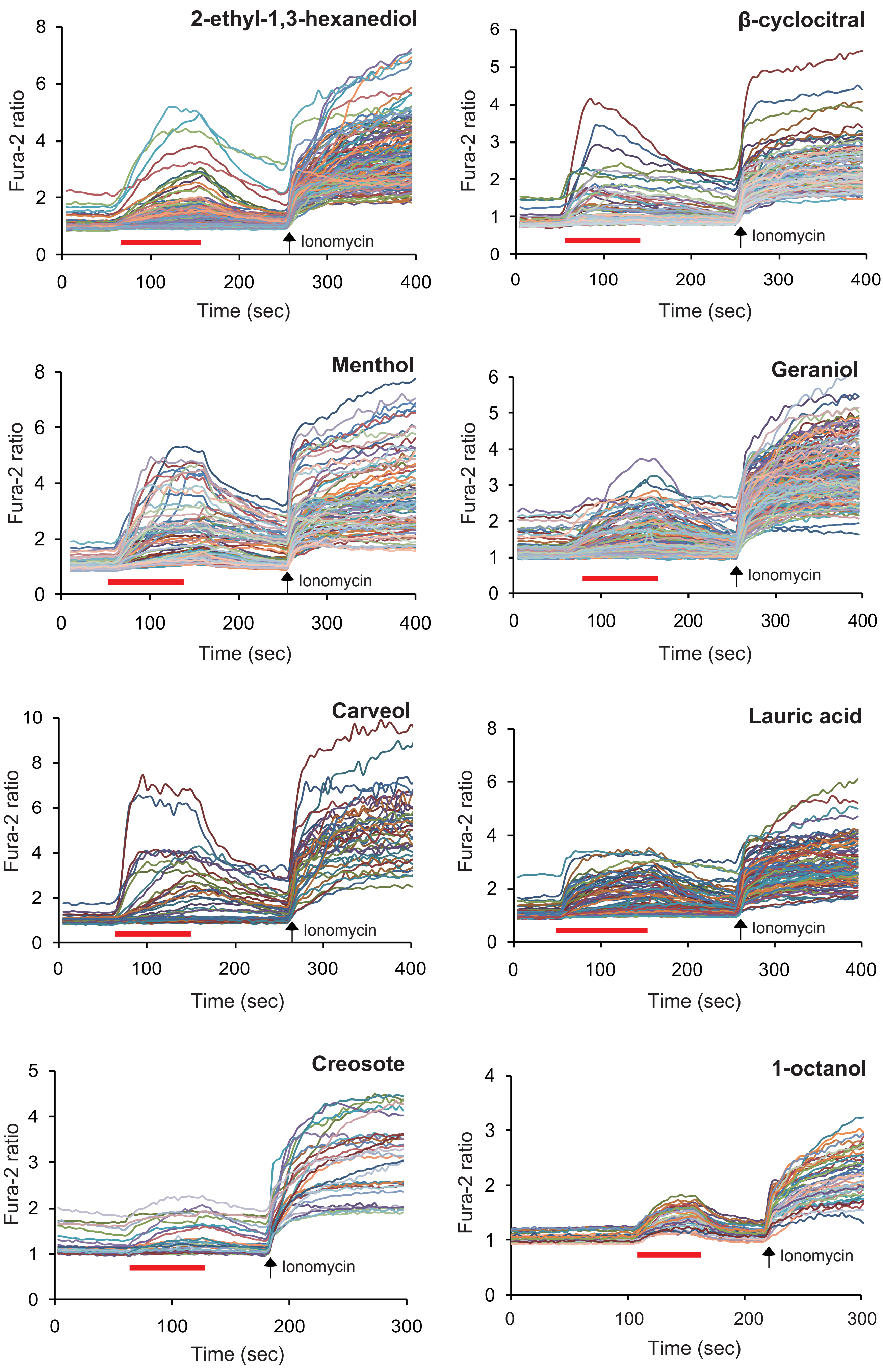

Supplementary figure 2d

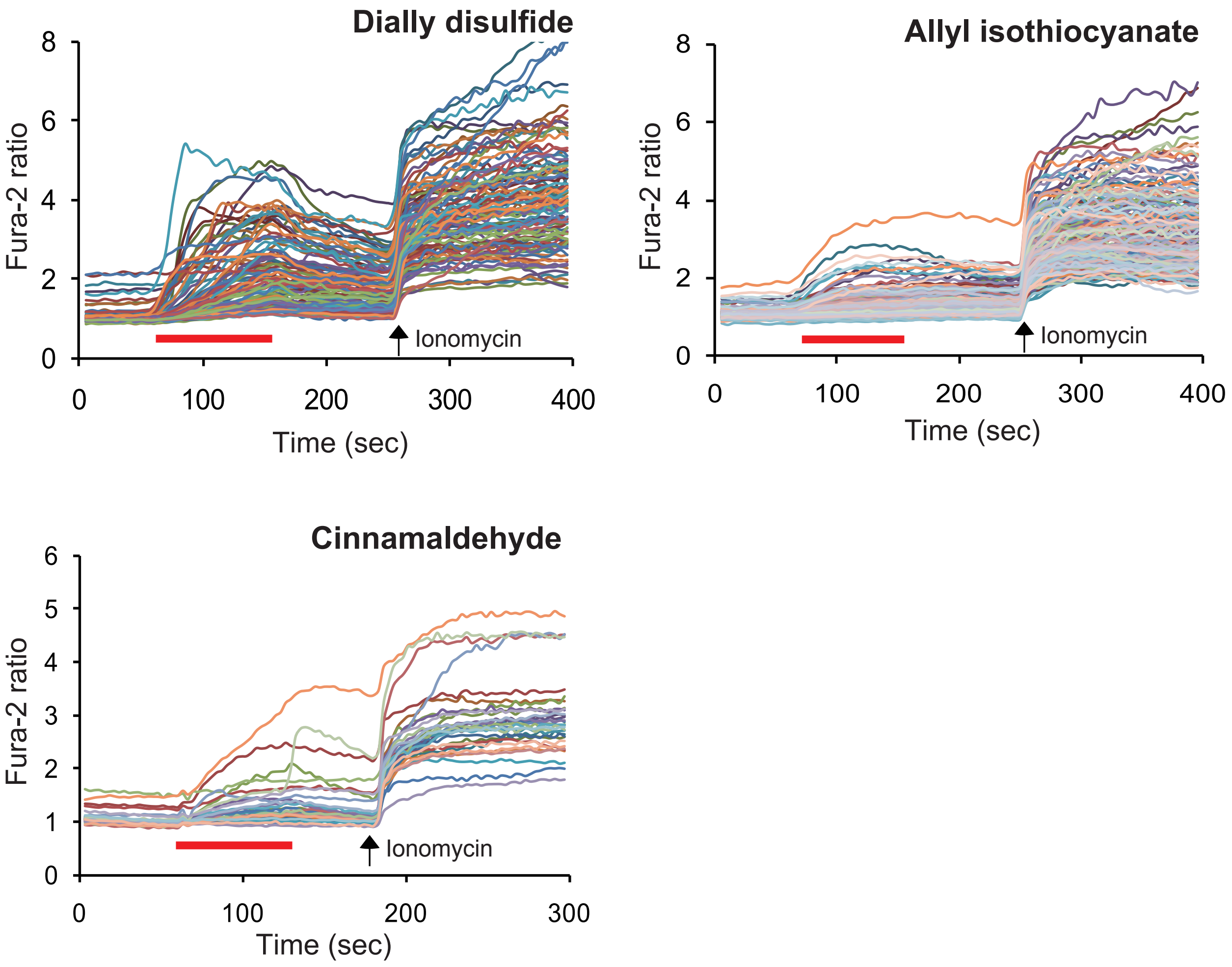

Supplementary figure 3

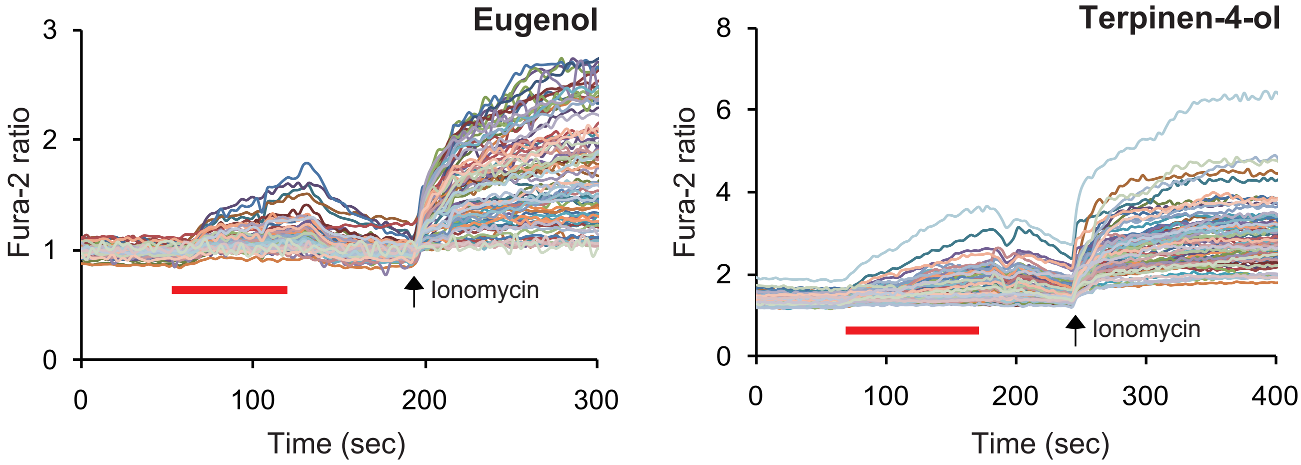

Supplementary figure 4

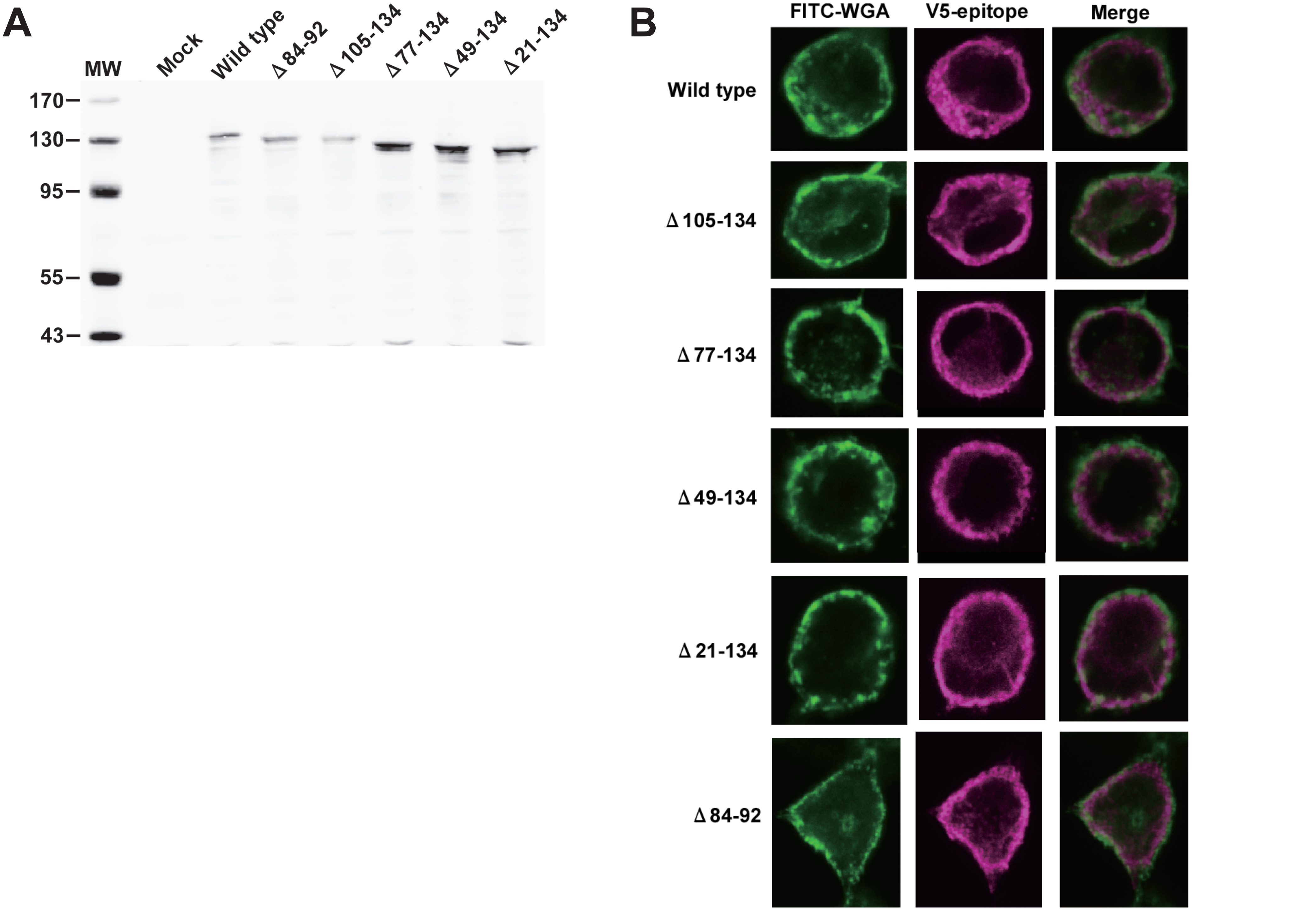

Supplement: Supplementary figures and legends, Table S1 [file rsob160042supp1.pdf]
